# Supplementary material for: Micromanipulation System for Isolating a Single Cryptosporidium Oocyst
Source: Micromachines (Basel). 2019 Dec 18;11(1):3. doi: 10.3390/mi11010003 (PMC7019727; doi:10.3390/mi11010003)
Supplement: Supplementary file 1 [file micromachines-11-00003-s001.zip › micromachines-11-00003-s001.pdf]

micromachines

Supplemenmtary

Micromanipulation System for Isolating a Single Cryptosporidium Oocyst

Hamish Penny [1], David T. S. Hayman [2] and Ebubekir Avci [1,*]

1 Department of Mechanical and Electrical Engineering, Massey University, Palmerston North, 4410, New Zealand; hamishrpenny@hotmail.com
2 School of Veterinary Science, Massey University, Palmerston North, 4410, New Zealand; D.T.S.Hayman@massey.ac.nz
* Correspondence: E.Avci@massey.ac.nz

Explanation S1: Accuracy calculation

To test the accuracy of the chosen alignment strategy an experiment was conducted using two end effectors, with the second "passive" end effector placed at a known angle relative to the end effector being aligned. To begin, an additional z axis actuator was used to manually align the passive end effector with the focus plane. The "active" end effector being tested was aligned with the focus plane using the automated alignment method, then actuated along the y axis in increments of 1μm until interference between the two end effectors was observed. The accuracy of the initial manual alignment of the passive end effector was checked in the experiment by ensuring the alignment error did not favour a positive or negative value. The method to check this was to move the active end effector vertically upwards after the experiment and observe if there was increased movement of the passive end effector, in which case it was assumed the alignment error had been negative.

To calculate the alignment error a mathematical model was developed. The following notation is used:

- E–Error of the alignment.
- σ–The taper of the end effectors (the difference in angle between the top and bottom surfaces).
- α–The angle difference between the centroid of the end effectors superimposed on the horizontal plane.
- d–Distance from each end effector tip to the point of first contact between the end effectors.
- a–The y-axis distance between the center of each end effector tip.
- b–The distance between the external edge of the tips of each end effector (assuming the tip has radius r).

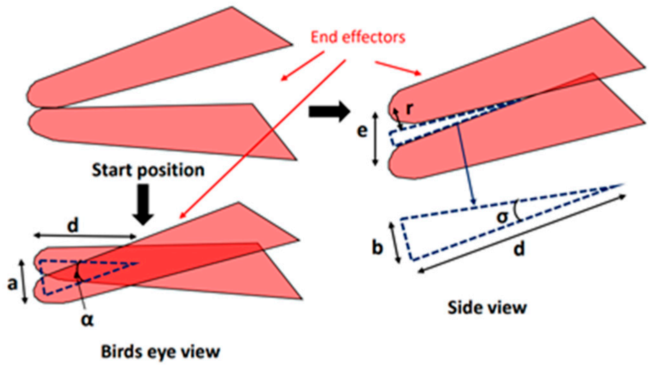

Figure S1. Relative positions of two end effectors

Notation used for calculation of the accuracy of the alignment experiment.

Trigonometry was used to find the relationship between the observed overlap of the end effectors (a) and the error in alignment between the end effectors (e). The following assumptions are used to simplify the analysis:

- The d value from each perspective is equal–this means the point of first contact would lie at the vertical maximum or minimum of each end effector. This is valid when angles σ and α are small.
- The error (e) is assumed to equal $b + 2r$. This is valid when the angle between the end effectors and the horizontal plane is small.

Based on these assumptions the relationship between the error (e) and displacement (a) is:

$$e = a\left(\frac{sin\frac{\sigma}{2}}{sin\frac{\alpha}{2}}\right) + 2r$$

© 2019 by the authors. Licensee MDPI, Basel, Switzerland. This article is an open access article distributed under the terms and conditions of the Creative Commons Attribution (CC BY) license (http://creativecommons.org/licenses/by/4.0/).
